# The effectiveness of fermented turmeric powder in subjects with elevated alanine transaminase levels: a randomised controlled study

**DOI:** 10.1186/1472-6882-13-58

**Published:** 2013-03-08

**Authors:** Sang-Wook Kim, Ki-Chan Ha, Eun-Kyung Choi, Su-Young Jung, Min-Gul Kim, Dae-Young Kwon, Hye-Jung Yang, Min-Jung Kim, Hee-Joo Kang, Hyang-Im Back, Sun-Young Kim, Soo-Hyun Park, Hum-Young Baek, Yong-Jae Kim, Joon-Yeol Lee, Soo-Wan Chae

**Affiliations:** 1 Clinical Trial Center for Functional Foods, Chonbuk National University Hospital, 560-182, Jeonju, Chonbuk, South Korea; 2 Department of Internal Medicine and Research Institute of Clinical Medicine, Chonbuk National University Hospital, Jeonju, Republic of Korea; 3 Department of Medical Nutrition Therapy, Chonbuk National University, Jeonju, Republic of Korea; 4 Healthcare Claims & Management Incorporation, Jeonju, Republic of Korea; 5 Korea Food Research Institute, Baekhyeon-dong, Bundang-gu, Seongnam-City, Gyeonggi-do, Republic of Korea; 6 Korea INS Pharmaceutical Company, Daepo-ri, Dong-myeon, Hwasoon-gun, Jeollanam-do, Republic of Korea

## Abstract

**Background:**

Previous animal studies have shown that *Curcuma longa* (turmeric) improves liver function. Turmeric may thus be a promising ingredient in functional foods aimed at improving liver function. The purpose of the study is to investigate the hepatoprotective effect of fermented turmeric powder (FTP) on liver function in subjects with elevated alanine transaminase (ALT) levels.

**Methods:**

A randomised, double-blind, placebo-controlled trial was conducted between November 2010 and April 2012 at the clinical trial center for functional foods of the Chonbuk National University Hospital. The trial included 60 subjects, 20 years old and above, who were diagnosed mild to moderate elevated ALT levels between 40 IU/L and 200 IU/L. Sixty subjects were randomised to receive FTP 3.0 g per day or placebo 3.0 g per day for 12 weeks. The treatment group received two capsules of FTP three times a day after meals, for 12 weeks. The primary efficacy endpoint was change in the ALT levels in the two groups. The secondary efficacy endpoints included its effect on aspartate aminotransferase (AST), gamma-glutamyl transferase (GGT), total bilirubin (TB), and lipid profiles. Safety was assessed throughout the study using ongoing laboratory tests. Adverse events (AEs) were also recorded.

**Results:**

Sixty subjects were randomised in the study (30 into the FTP group, 30 into the placebo group), and among them, twelve subjects were excluded from the analysis for protocol violation, adverse events or consent withdrawal. The two groups did not differ in baseline characteristics. After 12 weeks of treatment, 48 subjects were evaluated. Of the 48 subjects, 26 randomly received FTP capsules and 22 received placebo. The FTP group showed a significant reduction in ALT levels after 12 weeks of treatment compared with the placebo group (*p* = 0.019). There was also observed that the serum AST levels were significantly reduce in the FTP group than placebo group (*p* = 0.02). The GGT levels showed a tendency to decrease, while the serum alkaline phosphatase (ALP), TB, and lipids levels were not modified. There were no reported severe AEs during this study, or abnormalities observed on blood glucose, total protein, albumin, blood urea nitrogen (BUN), and creatinine levels.

**Conclusion:**

The data of this trial indicate that FTP is effective and safe, generally well-tolerated without severe AEs, in the treatment of subjects with elevated ALT levels over a 12 weeks period.

**Trial registration:**

ClinicalTrials.gov: http://NCT01634256

## Background

The liver plays a major role in detoxification and excretion of many endogenous and exogenous compounds. Any impairment to its functions may lead to many implications on one’s health. Management of liver diseases is still a challenge to the modern scientific community [1]. There are few conventional drugs that can stimulate liver function and offer hepatoprotection or help in the regeneration of hepatic cells [2]. Many plant-derived natural products have the potential to be hepatoprotective and therefore can be used to treat acute and chronic liver diseases [3-6]. The hepatoprotective activity of turmeric (*Curcuma longa*) or its constituent are reported in the literature [7-12].

Turmeric is a perennial herb of the ginger family, with significant antioxidant activity comparable to standard antioxidant such as butylated hydroxyanisole (BHA) [13]. It can grow up to 1 m high, and has oblong, tufted leaves. The powder of dried roots and rhizomes of turmeric are used as one of the spices in Indian curries and other cuisine. The three major components in turmeric are curcumin, demethoxycurcumin (DMC) and bisdemethoxy-curcumin (BDMC) [14].

Numerous studies have shown that curcumin has antioxidant and anti-inflammatory properties [15-17]. Although curcumin has been used throughout history, especially in India and Asia, the first study on curcumin and its dose-limiting toxicity was not published until 2001, when it was reported that amounts of up to 8 g, administered per day for three months, were not toxic to humans [18]. Recent evidence has shown that turmeric or curcumin improve the liver function in rats with hepatic injury model [7,9-12,19-24].

In view of the hepatoprotective and other beneficial effects of FTP in animal models, a randomized, double-blind, placebo-controlled clinical trial was designed and conducted to evaluate the effects of 12-week FTP treatment on serum aminotransferase levels in subjects with elevated ALT levels. A significant and clinically relevant improvement in these parameters would provide a solid rationale for performing a subsequent trial testing histological improvement.

## Methods

### Study design and ethics approval

This study was a single-center, randomised, double-blind, placebo-controlled clinical trial to evaluate the safety and efficacy of FTP in subjects with elevated ALT levels in Korea. The principal investigator (PI) initially screened each potential participant against the inclusion and exclusion criteria. All participants were screened and evaluated the hepatic ultrasonography, liver function test parameters (ALT, aspartate aminotransferase (AST), gamma-glutamyl transferase (GGT), total protein, serum albumin, and total bilirubin (TB)), lipid profiles (total cholesterol (TC), triglyceride (TG), high density lipoprotein-cholesterol (HDL-C), and low density lipoprotein-cholesterol (LDL-C)), and viral hepatitis test (hepatitis B virus (HBV) surface antigen and hepatitis C virus (HCV) antibody). After completing a screening test, eligible subjects were randomised to either the FTP or the placebo group. A total of 60 healthy subjects [56 males, 4 females; (mean ± SD) age: 37.6 ± 8.06 y; body mass index (BMI): 27.1 ± 3.4 kg/m^2^] were included in this study. All subjects were recruited from the population of Jeonju-city in Korea. The study was conducted in accordance with the Declaration of Helsinki, and written informed consent was obtained from each participant before allocation. To avoid allocation bias, concealed allocation using a sealed envelope was employed in this study. A statistician randomized participants using computer-generated random table in a 1:1 ratio with block size 2, and clinical research coordinators (CRC) assigned them by the random table to receive FTP or placebo treatment. The study was approved by the Functional Foods Institutional Review Board of Chonbuk National University Hospital (CUH IRB 2010-02-017), Jeonju, South Korea, where the study took place.

### Inclusion/exclusion criteria

All the subjects enrolled in the study should meet the inclusion criteria. The criteria mainly include: (1) age between 20 and 70 years; (2) ALT levels >40 IU/L measured at screening in the study laboratory; (3) readiness to comply with randomisation, treatment and follow-up; (4) subjects giving written informed consent. Subjects were excluded if they fulfilled any of the following criteria: (1) any laboratory (serologies for HBV and HCV); (2) abnormal transferrin saturation; (3) decompensated cirrhosis; (4) serious disease limiting life expectancy; (5) pregnancy and lactation.

### Study medication

The duration of treatment was 12 weeks, with either FTP (3.0 g) or placebo (3.0 g) both administered as two capsules, three times a day orally after meals. Capsules containing 470 mg lactose and Gardenia yellow color, or 500 mg FTP, were manufactured by Korea INS Pharmaceuticals, Inc. (Hwasoon, Jeonnam, South Korea). In Brief, crushed turmeric was fermented with 2% (wt/wt) of *Aspergillus oryzae* at 25°C for 36 h and dried.

We chose single doses to approximate the previously reported doses from hepatoprotective animal experiment [23]. Fermented turmeric was standardized to 0.79 mg curcumin per 1.0 g powder.

Curcumin content of the trial FTP was analysed by the Korea health supplement Institute using the high performance liquid chromatography (HPLC) assay. The amount of curcumin in the FTP was determined from calibration curve obtained by the concentration of curcumin standard against the peak area. Average curcumin contents in non-fermented turmeric and FTP were approximately 2.0 mg/g and 0.79 mg/g, respectively. The medication was delivered by the pharmacy of the Clinical Trial Center for Functional Foods in the Chonbuk National University Hospital and prescribed regularly to the subjects. The pharmacy established before the start of the study a list randomly assigning each subject to 1 of the 2 arms of the study. The subjects as well as investigators were blinded to the treatment until completion of the whole study. In this study, the subjects were not actively asked to change their lifestyle or to change their diet.

### Study end points

The objective of this randomised controlled trial was to investigate the effects of FTP on serum ALT levels, because the hypothesis tested was that FTP might improve liver function. Therefore, serum ALT levels was defined as the primary end point of the study. Secondary end points were changes in serum AST, GGT, ALP, and TB levels. The safety associated with treatment was also assessed by laboratory tests including glucose, total protein, albumin, BUN, and creatinine levels. Blood samples for the assessment of clinical outcomes were obtained at week 0 (baseline), 6 and 12. The AEs were recorded at every visit. The clinical laboratory assessments (hematology, clinical biochemistry and urinalysis) were performed at intervals throughout the study. The HBV, HCV, and liver ultrasounds were also performed at screening visit. Blood pressure, weight and abdominal circumference were measured every 6 week by a trained research nurse. The questionnaires assessing physical activity, smoking status, alcohol intake, and general health were administered at baseline and after each intervention period (at week 6 and 12).

### Statistical analysis

For sample size calculation, there was no previous clinical trial to compare the FTP with placebo, therefore, this study was designed as a pilot study to calculate the appropriate sample size for future rigorous randomized clinical trials. We assumed that the primary outcome was serum ALT levels, whereby 10.2 IU/L was expected a clinically relevant difference between the two groups, with a standard deviation of 14.1, alpha set on 5% and power on 80%. This resulted in a required number of 24 subjects in each group. With an estimated 20% dropout rate, we set the total sample size at 60.

All data were entered into a data sheet twice and reviewed to ensure accuracy. The analyst was blinded to group allocation. The primary efficacy outcome was analysed in the modified per-protocol (PP) population, who completed the study without any major protocol violation. The assessments of secondary efficacy outcomes, including serum AST, ALP, GGT, and TB, were primarily based on the PP population. Differences between treatment groups were compared using Linear mixed model for repeated measures data or *t*-test for continuous variables and chi-square or Fisher’s exact tests for categorical variables.

All statistical calculations were performed using SAS for Windows software (version 9.2, SAS institute, Inc., Cary, NC, USA), and the level of significance was established at *p* = 0.05.

## Results

### Subject disposition and baseline characteristics

**Figure 1:**
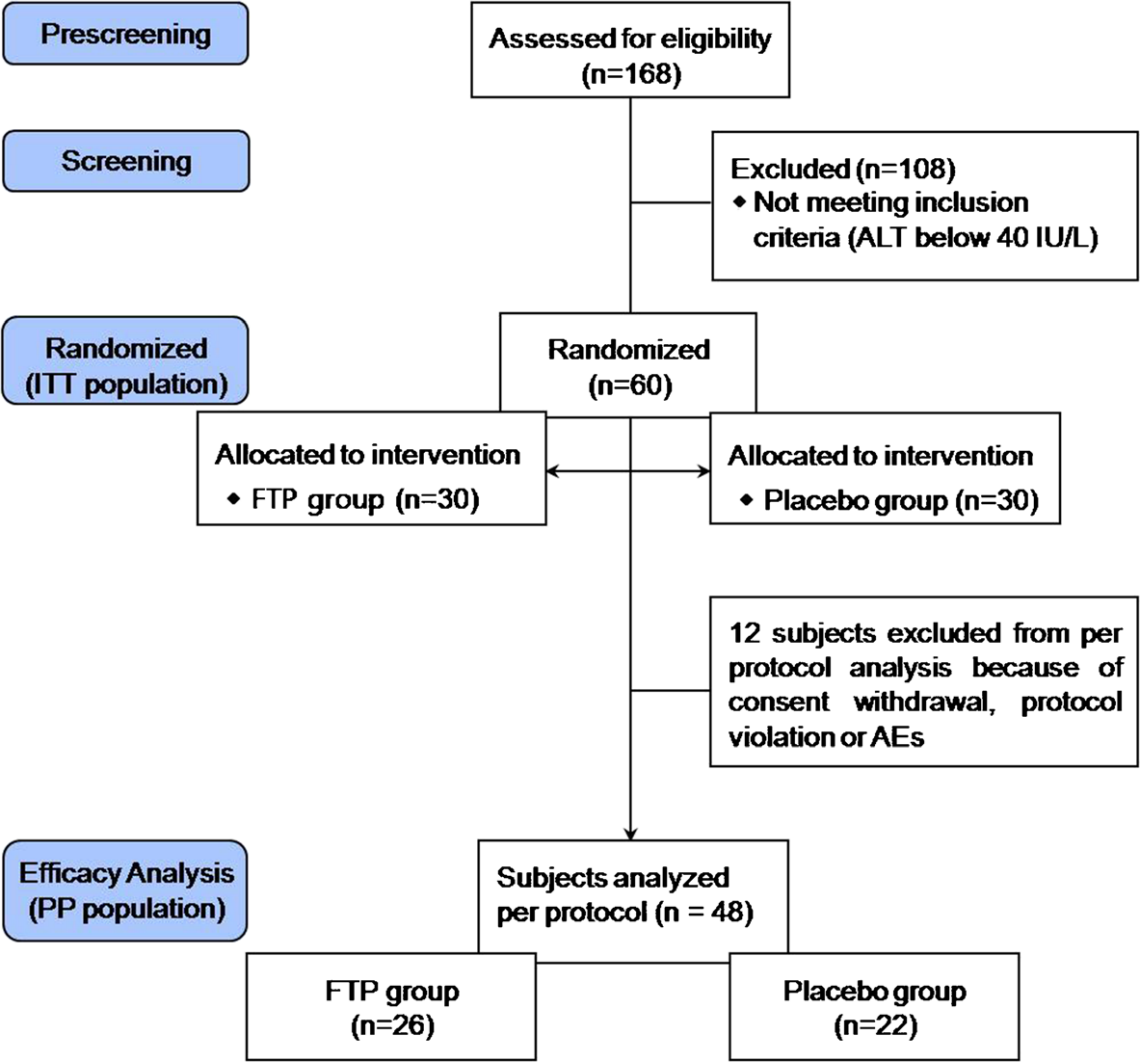
**Flow chart for the study subjects.** AEs, adverse events; ALT, alanine aminotransferase; FTP, fermented turmeric powder; ITT, intent-to-treat; PP, per protocol.

Figure 1 shows the flow chart of subject participation in the trial. A total of 168 subjects were screened, and 60 subjects were randomised (30 in the FTP group and 30 in the placebo group). Of 60 randomised subjects, 1 withdrew consent during the study and 3 discontinued treatment because of protocol violation (use of prohibited concomitant medication). Of the 56 subjects, eight subjects were excluded from the efficacy analysis because of protocol violation or AEs (abnormal values). Thus, 48 subjects were included in the PP analysis (n = 26 for FTP, n = 22 for placebo) (12 subjects were excluded from the PP analysis due to consent withdrawal, protocol violation or AEs; 4 subjects in the FTP group and 8 subjects in the placebo group). The overall compliance in this study was 80%.

Baseline subject characteristics are shown in Table 1. There were no significant differences between the two groups. In none of the 2 groups the BMI changed significantly during the 12 week, and the mean BMI remained similar between the 2 groups until completion of the study. Most subjects were asymptomatic at inclusion and remained so throughout the course of the study. Throughout the study period, no significant lifestyle changes were reported in either treatment group. Alcohol consumption remained stable throughout the study period in both groups. Although both kinds of capsules appeared identical, it is possible that some of the subjects could discern differences between them.

**Table 1:** Baseline characteristics of the study subjects

	**FTP group (n = 30)**	**Placebo group (n = 30)**	**Total (n = 60)**	***P*-value**^**1)**^
Sex(M/F)	28/2	28/2	56/4	1.000^2)^
Age (years)	39.0 ± 8.5	36.2 ± 7.4	37.6 ± 8.1	0.170
Height (cm)	171.1 ± 6.1	170.7 ± 5.8	170.9 ± 5.9	0.770
Weight (kg)	78.5 ± 10.9	79.6 ± 10.0	79.1 ± 10.4	0.674
BMI (kg/m^2^)	26.8 ± 3.3	27.4 ± 3.5	27.1 ± 3.4	0.512
SBP (mmHg)	126.6 ± 11.2	129.2 ± 10.3	127.9 ± 10.8	0.341
DBP (mmHg)	79.6 ± 9.3	79.8 ± 8.5	79.7 ± 8.8	0.931
Pulse (BPM)	76.4 ± 12.2	75.7 ± 7.9	76.1 ± 10.2	0.783

Abbreviation: M, male; F, female; SBP, systolic blood pressure; DBP, diastolic blood pressure; BPM, beats per minute.

Values are presented as mean ± SD.

^1)^ Analyzed by Independent *t*-test.

^2)^ Analyzed by Fisher's exact-test.

### Efficacy of treatment

**Figure 2:**
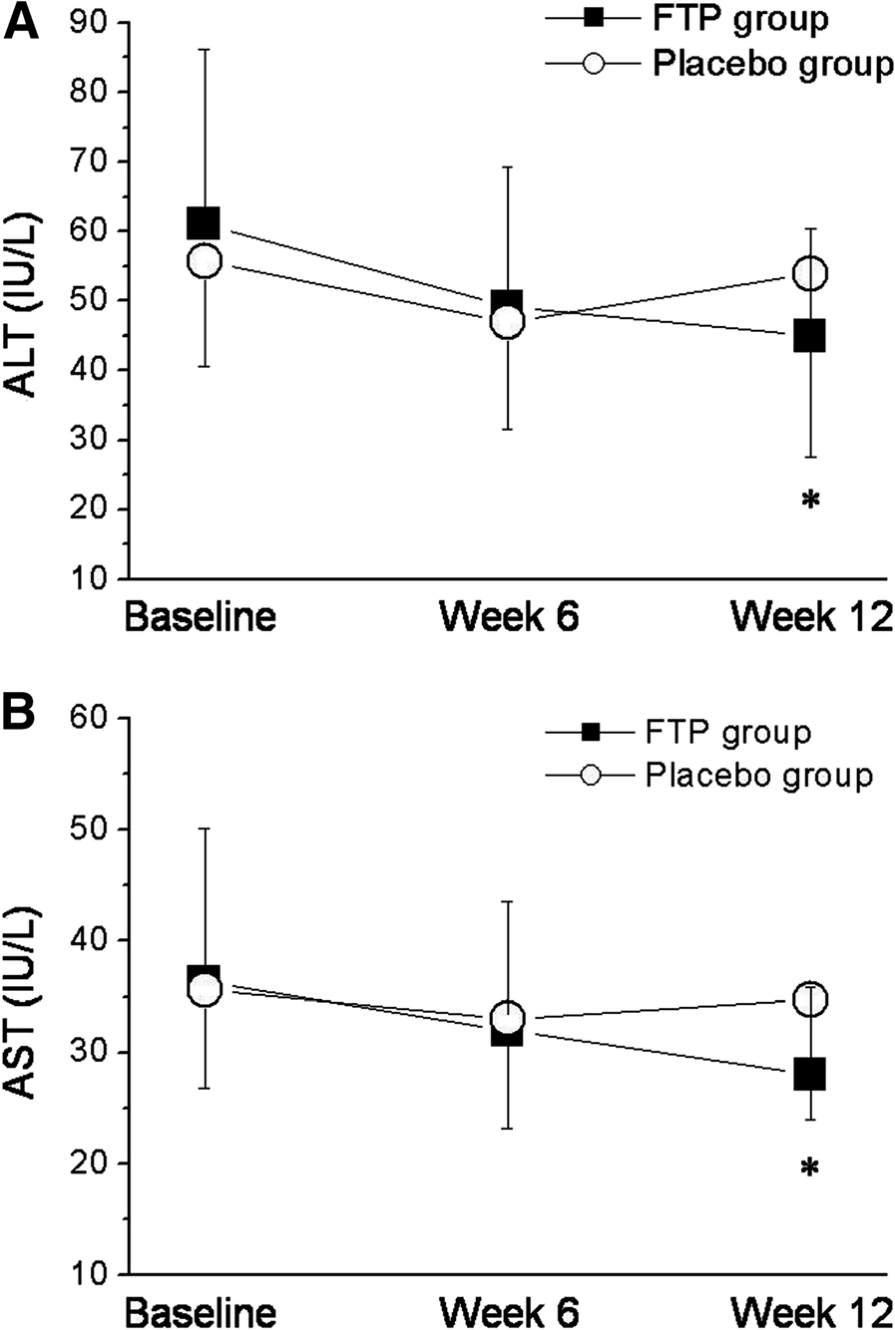
**Effects on serum aminotransferase levels.** (***A***) Evolution of ALT during the study period. *Asterisks* indicate a difference in comparison to value at inclusion (^*^p = 0.019). (***B***) Evolution of AST during the study period. *Asterisks* indicate a difference in comparison to value at inclusion (^*^p = 0.020).

Treatment with FTP was associated with a decline in both ALT and AST levels. These reductions were apparent after 6 week of treatment and were maintained throughout the 12-week double-blind treatment period in FTP treatment groups (Figure 2). The declines in both ALT and AST observed at week 12 were statistically significant compared with placebo.

**Figure 3:**
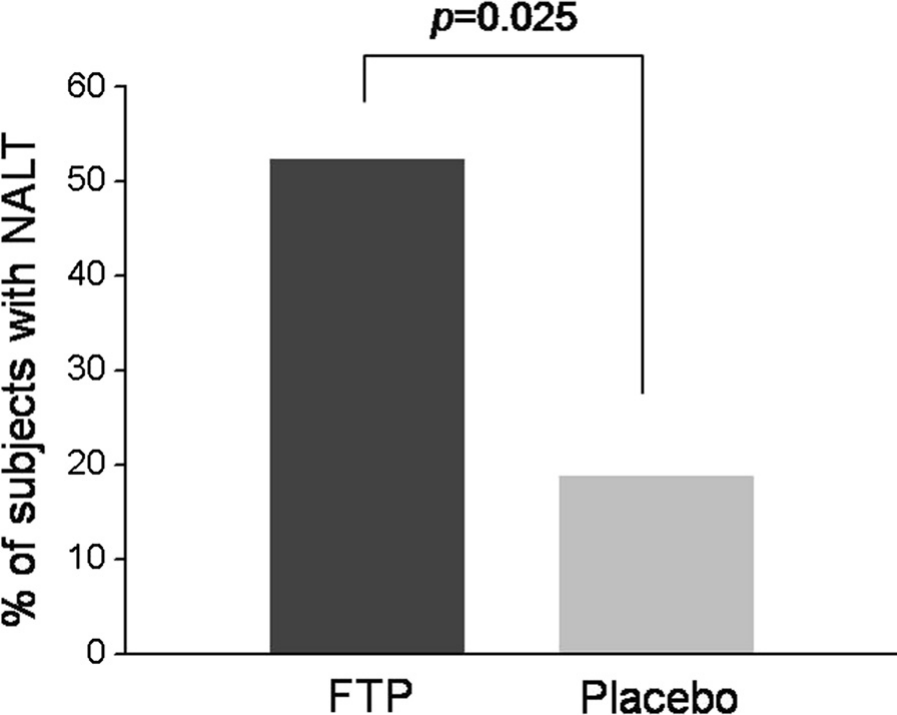
**Proportion of subjects with ALT normalization at end of treatment (week 12).** NALT, normalized alanine aminotransferase.

The complete data set for all measured parameters is listed in Table 2. At week 12, the mean ALT reduction was significantly greater in the FTP group than in the placebo group (−26.5% vs. -3.4%; *p =* 0.019). There was also observed that the mean AST levels were significantly reduce in the FTP group than placebo group (−23.1% vs. -2.2%; *p* = 0.020). The mean GGT reduction was greater in the FTP group than in the placebo group, but this reduction did not reach statistical significance (−22.5% vs. -3.4%; (*p =* 0.254). The ALP and total bilirubin levels were not significantly affected. Figure 3 shows the proportion of subjects with ALT normalization at week 12. The ALT normalization rate was significantly higher in the FTP group than in the placebo group (*p* = 0.025).

**Table 2:** Biochemical parameters at baseline (week 0) and end of treatment (week 12) with FTP or placebo in subjects with elevated ALT levels

	**FTP group (n = 26)**		**Placebo group (n = 22)**		***P*-value**^**1)**^
	**Baseline**	**12 week**	**Baseline**	**12 week**	
ALT (IU/L)					
Mean ± SD	61.1 ± 25.1	44.9 ± 15.5	55.7 ± 15.1	53.8 ± 26.3	0.019^*^
Median(Range)	51.5(41–151)	42.5(19–95)	49(40–85)	51(22–127)	-
AST (IU/L)					
Mean ± SD	36.3 ± 13.8	27.9 ± 7.9	35.6 ± 8.8	34.8 ± 10.9	0.020^*^
Median(Range)	32.5(20–73)	27(17–46)	34(25–57)	33.5(21–55)	-
ALP (IU/L)					
Mean ± SD	85.0 ± 19.3	80.0 ± 21.9	71.7 ± 13.5	68.9 ± 13.2	0.471
Median(Range)	81.5(52–144)	79(46–160)	69(48–101)	69(43–92)	-
gamma-GT (IU/L)					
Mean ± SD	86.3 ± 71.5	66.9 ± 45.6	104.3 ± 69.0	100.8 ± 68.4	0.254
Median(Range)	64(26–360)	52(23–195)	95(16–231)	91(17–253)	-
Total bilirubin (mg/dL)					
Mean ± SD	0.98 ± 0.46	0.88 ± 0.38	0.89 ± 0.28	0.84 ± 0.26	0.664
Median(Range)	0.87(0.37-2.29)	0.79(0.4-1.98)	0.9(0.37-1.37)	0.81(0.37-1.34)	-

Values are presented as mean ± SD.

1. Linear mixed model for repeated measures data.

^*^P <0.05.

### Safety

 Results of laboratory testing performed during the study are shown in Table 3. Laboratory values obtained at baseline and after completing FTP or placebo treatment were within acceptable range. There were no significant difference in any of the blood chemistry parameters among treatment groups. There were no serious AEs reported during the study period. Five participants reported AEs during the study. Two subjects in the Placebo group experienced mild enteritis and ureterolith, respectively. Three subjects in the FTP group had chronic gastritis, gout-induced pain, and common cold, respectively. These five reports, as judged by the PI, were unlikely related to the study protocol. These AEs were mild and were pre-existing complaints before the initiation of treatment with FTP or placebo. No significant differences in AEs were found between the two groups.

**Table 3:** The laboratory findings for safety after the duration of the clinical trial

	**Turmeric group (n = 26)**		**Placebo group (n = 22)**		***P*-value**^**1)**^
	**Baseline**	**12 week**	**Baseline**	**12 week**	
WBC (×10^3^/μl)	6.8 ± 1.7	6.7 ± 1.7	6.2 ± 1.4	6.2 ± 1.4	0.79
RBC (×100^3^/μl)	5.1 ± 0.3	5.1 ± 0.4	5.0 ± 0.3	5.0 ± 0.3	0.36
Hemoglobin (g/dL)	15.9 ± 1.1	15.9 ± 1.2	15.6 ± 1.4	15.7 ± 1.3	0.21
Hematocrit (%)	45.9 ± 2.9	46.0 ± 3.4	44.8 ± 2.9	45.4 ± 3.0	0.37
Platelet (×10^3^/μl)	252.2 ± 49.3	250.3 ± 44.3	255.6 ± 58.5	254.5 ± 62.7	0.92
Total cholesterol (mg/dl)	193.6 ± 25.2	201.7 ± 30.0	194.2 ± 34.7	198.6 ± 31.7	0.62
Triglyceride (mg/dL)	213.3 ± 114.4	220.9 ± 132.8	172.8 ± 78.0	209.4 ± 167.6	0.44
HDL-cholesterol (mg/dL)	44.1 ± 11.8	43.69 ± 11.2	45.8 ± 10.2	48.5 ± 12.1	0.16
LDL-cholesterol (mg/dL)	122.5 ± 29.4	117.3 ± 27.6	125.9 ± 38.9	113.6 ± 27.5	0.33
Glucose (mg/dL)	91.9 ± 9.7	93.0 ± 12.6	90.1 ± 8.7	89.6 ± 8.7	0.52
Total Protein (g/dL)	7.4 ± 0.3	7.48 ± 0.4	7.3 ± 0.4	7.4 ± 0.4	0.73
Albumin (g/dL)	4.6 ± 0.2	4.63 ± 0.2	4.57 ± 0.2	4.6 ± 0.3	0.79
BUN (mg/dL)	14.5 ± 4.7	13.5 ± 3.6	13.3 ± 2.6	12.9 ± 2.8	0.58
Creatinine (mg/dL)	0.85 ± 0.13	0.81 ± 0.14	0.81 ± 0.14	0.83 ± 0.14	0.12

Values are presented as mean ± SD.

^1)^ Linear mixed model for repeated measures data.

## Discussion

The aim of this study was to evaluate the effects of FTP on liver function evaluating by aminotransferase levels in subjects with elevated ALT levels. The results show that the ingestion of 3.0 g FTP reduced serum aminotransferase levels in this randomised, double-blind placebo-controlled trial. In a previous study, reduction in ALT and AST levels has been observed when 300 mg/kg fermented turmeric was administered to galactosamine-intoxicated rats daily for two weeks [23]. In a similar studies on galactosamine-or carbon tetrachloride-intoxicated animals, the serum ALT and AST levels were significantly attenuated in animals pretreated with turmeric extract or fermented turmeric [8,24]. In an *in vitro* study in which the effect of curcumin, the principal active compound of turmeric, on tacrine-induced cytotoxicity in human liver-derived Hep G2 cells was investigated, curcumin exhibited hepatoprotective activity [25].

 The effectiveness of FTP on hepatic function has not been studied previously in humans. Liver enzymes ALT and AST are often used as markers of hepatic function and their increase in blood indicates liver damage. It is tempting to speculate that reduction in ALT and AST levels reflects improvement in hepatic function. Therefore, data from the current trial, showing a reduction of ALT and AST levels, suggest that turmeric may improve hepatic function. This appears biologically plausible as several *in vivo* and *in vitro* actions of turmeric or curcumin are consistent with hepatoprotective actions. One of the most known mechanisms underlying turmeric’s beneficial effects on hepatic function is its pleiotropic anti-oxidant activity [26]. It prevents formation and scavenges reactive oxygen species (ROS) [27,28] and reactive nitrogen species [29,30]. Furthermore, curcumin was shown to induce several enzymatic anti-oxidants, like glutathione transferase [31], heme-oxygenase-1 [32] and catalase [31]. Curcumin inhibits the activation of NF-kB by ROS, protein kinase C (PKC) and pro-inflammatory cytokines. NF-kB-mediated expression of inducible nitric oxide synthase (iNOS) is also inhibited by curcumin [33,34].

 In the present study, we measured after 6 weeks and 12 weeks of administration of FTP. The results of our study show that the ingestion of 3.0 g FTP significantly reduced serum ALT and AST levels without severe AEs, indicating that turmeric may help improve hepatic disorders. These findings agree with experimental studies, which have found that FTP contained with curcumin reduces serum ALT and AST levels [23,35]. However, a significant reduction in ALP and total bilirubin levels after supplementation with FTP was not observed in our study, which can be attributed to the fact that the ALP and total bilirubin values at baseline in most of our subjects were in the normal range.

 Although the present study has produced promising results, subject’s health condition (borderline between healthy person and patient) and small sample size in this study limits the generalization of our results to other populations with hepatic disorders. Another limitation is that we assessed liver state only by measuring liver enzymes and did not perform computed tomography scans, MR scans, or liver biopsies. Finally, the present study was performed to Korean Food and Drug Administration (KFDA) approval for health functional food. For this reason, patients with chronic liver disease were excluded from participating in this study. Therefore, more rigorous investigations are needed to elucidate the hepatoprotective effects of FTP on hepatic disorders.

## Conclusion

In conclusion, FTP significantly reduced serum AST and ALT levels in subjects with elevated ALT levels. These effects were maintained for as long as subjects remained on treatment. FTP was well tolerated and without significant AEs. Further studies in larger numbers of subjects, for a longer period of time and with histologic endpoints, are required to determine whether FTP have protective beneficial effect on hepatic disorder.

## Competing interests

The authors declare that they have no competing interests.

## Authors’ contributions

SWK, SWC, HIB, EKC, and KCH planned the study design and conducted the study. SYJ, MGK, SYK, and SHP performed the statistical planning and analyses of the study and wrote the final report. HJY, MJK, HJK, and JYL organized all the legal and administrative requirements to perform the study and contributed to the coordination of the study. DYK, HYB and YJK received the research funding. All authors read and approved the final manuscript.

## Pre-publication history

The pre-publication history for this paper can be accessed here:

http://www.biomedcentral.com/1472-6882/13/58/prepub
